# Traditional Chinese Medicine formula Dai-Zong-Fang alleviating hepatic steatosis in *db/db* mice via gut microbiota modulation

**DOI:** 10.3389/fphar.2024.1337057

**Published:** 2024-01-24

**Authors:** Li-Wei Zhang, Li-Li Zhu, Xiao-Yun Zhu, Shou-Qiang Fu, Xi-Ming Liu

**Affiliations:** ^1^ Department of Laboratory of Diabetes, Guang’anmen Hospital, China Academy of Chinese Medical Sciences, Beijing, China; ^2^ School of Life Science, Beijing University of Chinese Medicine, Beijing, China; ^3^ Pulmonary Disease Department of Integrated Traditional Chinese and Western Medicine, China-Japan Friendship Hospital, Beijing, China

**Keywords:** Dai-Zong-Fang, Traditional Chinese Medicine, gut microbiota, gut-liver axis, lipid absorption, intestinal barrier, hepatic lipid metabolism

## Abstract

**Introduction:** Hepatic steatosis is a hepatic pathological change closely associated with metabolic disorders, commonly observed in various metabolic diseases such as metabolic syndrome (MetS), with a high global prevalence. Dai-Zong-Fang (DZF), a traditional Chinese herbal formula, is widely used in clinical treatment for MetS, exhibiting multifaceted effects in reducing obesity and regulating blood glucose and lipids. This study aims to explore the mechanism by which DZF modulates the gut microbiota and reduces hepatic steatosis based on the gut-liver axis.

**Methods:** This study utilized *db/db* mice as a disease model for drug intervention. Body weight and fasting blood glucose were monitored. Serum lipid and transaminase levels were measured. Insulin tolerance test was conducted to assess insulin sensitivity. Hematoxylin and eosin (HE) staining was employed to observe morphological changes in the liver and intestine. The degree of hepatic steatosis was evaluated through Oil Red O staining and hepatic lipid determination. Changes in gut microbiota were assessed using 16S rRNA gene sequencing. Serum lipopolysaccharide (LPS) levels were measured by ELISA. The expression levels of intestinal tight junction proteins, intestinal lipid absorption-related proteins, and key proteins in hepatic lipid metabolism were examined through Western blot and RT-qPCR.

**Results:** After DZF intervention, there was a decrease in body weight, alleviation of glucose and lipid metabolism disorders, reduction in serum aspartate aminotransferase (AST) and alanine aminotransferase (ALT) levels, and mitigation of insulin resistance in mice. DZF significantly modulated the diversity of the gut microbiota, with a notable increase in the abundance of the *Bacteroidetes* phylum. PICRUSt indicated that DZF influenced various functions in gut microbiota, including carbohydrate and amino acid metabolism. Following DZF intervention, serum LPS levels decreased, intestinal pathological damage was reduced, and the expression of intestinal tight junction protein occludin was increased, while the expression of intestinal lipid absorption-related proteins cluster of differentiation 36 (CD36) and apolipoprotein B48 (ApoB48) were decreased. In the liver, DZF intervention resulted in a reduction in hepatic steatosis and lipid droplets, accompanied by a decrease fatty acid synthase (FASN) and stearoyl-CoA desaturase 1 (SCD1) and fatty acid transport protein 2 (FATP2). Conversely, there was an increase in the expression of the fatty acid oxidation-related enzyme carnitine palmitoyltransferase-1𝛂 (CPT-1𝛂).

**Conclusion:** DZF can regulate the structure and function of the intestinal microbiota in *db/db* mice. This ameliorates intestinal barrier damage and the detrimental effects of endotoxemia on hepatic metabolism. DZF not only inhibits intestinal lipid absorption but also improves hepatic lipid metabolism from various aspects, including *de novo* lipogenesis, fatty acid uptake, and fatty acid oxidation. This suggests that DZF may act on the liver and intestine as target organs, exerting its effects by improving the intestinal microbiota and related barrier and lipid absorption functions, ultimately ameliorating hepatic steatosis and enhancing overall glucose and lipid metabolism.

## 1 Introduction

Influenced by unhealthy lifestyle habits such as a high-calorie diet and sedentary behavior, the global prevalence of obesity and metabolic syndrome (MetS) is rapidly increasing. MetS is characterized primarily by central obesity and abnormalities in lipid and glucose metabolism. If metabolic abnormalities continue to worsen, it will increase the risk of diseases such as type 2 diabetes, coronary heart disease, and malignant tumors (Hudish et al., 2019; Bishehsari et al., 2020; Stefan and Schulze, 2023). The liver plays a central and crucial role in whole-body glucose and lipid metabolism (Priest and Tontonoz, 2019). Pathological changes in the liver caused by metabolic abnormalities most commonly manifest as hepatic steatosis. Hepatic steatosis is considered a liver alteration in the context of MetS (Lim et al., 2021). It is characterized by an increased deposition of liver fat, which can aggregate into lipid droplets. Microscopically, large vacuolar fat changes can be observed (Powell et al., 2021). Hepatic steatosis exacerbates overall abnormalities in glucose and lipid metabolism and insulin resistance, creating a vicious cycle (Watt et al., 2019).

Hepatic steatosis is the collective result of multiple dysregulated lipid metabolic pathways, essentially characterized by an imbalance in the inflow and outflow of fatty acids within the liver. The liver has the capacity to both synthesize fatty acids *de novo* from scratch and uptake free fatty acids from the circulatory system (Nagarajan et al., 2022). From an initial external etiological perspective, unhealthy dietary habits and excessive energy intake lead to systemic energy overaccumulation, affecting the liver, and triggering obesity and hepatic steatosis. The intestine serves as the primary site for nutrient absorption and harbors a substantial microbial community that influences both intestinal and overall health (Wit et al., 2022). The intestine and the liver are directly anatomically connected through the portal vein, forming a complex network of interactions between the two in terms of nutrient absorption, metabolite generation, and microbial composition (Tilg et al., 2022). Imbalances in the ecology of the intestinal microbiota can lead to intestinal barrier disruption and pathogen translocation. Combined with alterations in intestinal lipid absorption function, these factors directly or indirectly influence liver metabolism. Investigating targets through the gut-liver axis for the treatment of metabolic diseases such as hepatic steatosis is a current hot topic in research (Martín-Mateos and Albillos, 2021).

Traditional Chinese Medicine (TCM) therapies have demonstrated certain advantages in improving metabolic diseases. TCM has shown certain advantages in the treatment of metabolic diseases. Dai-Zong-Fang (DZF) originates from *Xiaoxianxiong Decoction*, a renowned herbal formula documented in the classic Chinese medical text *Treatise on Cold Damage Diseases* (Zhang, 2005). It has long been used to treat conditions defined in TCM as “syndrome of combined phlegm and heat” or “thoracic accumulation” (Li et al., 2020), primarily encompassing modern medical conditions such as coronary heart disease and MetS (Han et al., 2018; Shen et al., 2022). Its herbal composition includes Coptis chinensis Franch. (Huanglian), Citrus×aurantium L. (Zhishi), Pinellia ternata (Thunb.) Makino (Banxia), Trichosanthes kirilowii Maxim (Gualou), Neolitsea cassia (L.) Kosterm. (Rougui) and red yeast rice (Hongqu). Studies have shown that the use of DZF in treating patients with MetS significantly reduces waist and hip circumferences and alleviates blood glucose and lipid abnormalities (Zhu et al., 2017). Furthermore, more in-depth research indicates that after intervention with DZF in mice subjected to diet-induced obesity C57BL/6J mice and *db/db* mice, it mitigates insulin resistance and disturbances in glucose and lipid metabolism (Zhu et al., 2018; Huang et al., 2022a). Additionally, DZF promotes browning of white adipocytes both *in vitro* and *in vivo* (Xu et al., 2023).

The gut is responsible for food digestion, absorption, and fecal elimination, and it harbors a diverse microbial community, serving as a critical barrier between the internal body environment and the external world. TCM, with a history spanning thousands of years, has long recognized the vital functions of the intestines and emphasized that the regulation of the intestines is predominantly governed by middle energizer, specifically the spleen and stomach. From the perspective of TCM theory, the core site of action for DZF is situated within middle energizer. Based on historical herbal texts, all six medicinal herbs in DZF are associated with the spleen or stomach meridians. Notably, Huanglian, Zhishi, Gualou, and Rougui all impact the large intestine meridian. These herbs are commonly employed in traditional herbal medicine for the treatment of gastrointestinal disorders such as abdominal pain, diarrhea, or constipation (Yang et al., 2021; Park et al., 2023). Berberine, extracted from Huanglian, is a natural compound extensively used in the management of intestinal diseases, primarily exerting anti-inflammatory and antioxidant effects (Zhu et al., 2022; Cao et al., 2023). The remaining five herbal ingredients have also demonstrated in various studies their capabilities in modulating gut microbiota, alleviating intestinal inflammation, and reducing intestinal permeability (Kim and Kim, 2019; Li et al., 2021b; Huang et al., 2022b; Bi et al., 2022; Su et al., 2022).

In our team’s previous research, it was observed that after DZF intervention, the serum non-esterified fatty acid levels decreased in *db/db* mice. Additionally, histological staining with HE and Oil Red O revealed a reduction in liver fat deposition. What specific upstream mechanisms does DZF employ to regulate liver lipid metabolism? Can the pharmacological mechanisms of DZF reflect the TCM explanations for DZF? Therefore, this study aims to delve deeper into the effects of DZF on hepatic steatosis from the perspective of the gut-liver axis.

## 2 Methods and materials

### 2.1 Chemicals and reagents

DZF is composed of six Chinese herbal medicines: *Coptis chinensis* Franch. (Huanglian), *Citrus×aurantium* L. (Zhishi), *Pinellia ternata* (Thunb.) Makino (Banxia), *Trichosanthes kirilowii* Maxim (Gualou), *Cinnamomum cassia* (L.) J. Presl (Rougui), and red yeast rice (Hongqu). DZF extract (freeze-dried powder) was produced and supplied by Zhejiang Jiu Xu Pharmaceutical Co., LTD. (Batch Number: YC20200501). The extraction method and quality control are detailed in the Chinese patent document titled “A Traditional Chinese Medicine Composition and Its Preparation for Treating Metabolic Syndrome” (Chinese Patent No: ZL201811080557.5) (Xu et al., 2023). High-performance liquid chromatography (HPLC) was used to determine the major active chemical components in DZF, including berberine, naringin, hesperidin, and lovastatin (Zhu et al., 2018). DZF freeze-dried powder was dissolved in ultrapure water with the assistance of ultrasonication to prepare the corresponding concentrations of DZF solution. Metformin hydrochloride tablets (Sino-American Shanghai Squibb Pharmaceuticals Ltd.) were crushed and dissolved in ultrapure water to prepare the corresponding concentrations of metformin solution. Both solutions were freshly prepared and used for animal experiments.

The primary antibodies used included: ZO-1 Antibody (#DF2250, Affinity, United States, 1:500), Occludin Antibody (#DF7540, Affinity, United States, 1:1000), APOB Polyclonal Antibody (20578-1-AP, Proteintech, United States, 1:1000), CD36 Polyclonal Antibody (18836-1-AP, Proteintech, United States, 1:500), FASN Polyclonal Antibody (10624-2-AP, Proteintech, United States, 1:5000), SCD Polyclonal Antibody (28678-1-AP, Proteintech, United States, 1:2000), CPT-1𝛂 Polyclonal Antibody (15184-1-AP, Proteintech, United States, 1:5000), FATP2 Polyclonal Antibody (14048-1-AP, Proteintech, United States, 1:2000), and β-Actin (13E5) (4970S, Cell Signaling, United States, 1:1000). Total cholesterol (TCH/T-CHO) measurement reagent kit (A11-1-1), triglyceride (TG) measurement reagent kit (A110-1-1), and total protein quantification measurement reagent kit (A045-4) were purchased from the Nanjing Jiancheng Bioengineering Research Institute (China). The Mouse Lipopolysaccharides ELISA Kit (CSB-E13066m) was obtained from CUSABIO. Glucose Oxidase Method Kit (E1010) was obtained from Applygen (China).

### 2.2 Animal experiments

Experimental mice used in this study included C57BL/Ksj-Lepr *db/db* mice with leptin receptor deficiency as the test subjects and their littermates, heterozygous *db/m* mice, as the normal controls. A total of 36 *db/db* mice and 12 *db/m* mice, all males and 6 weeks old, were obtained from Changzhou Cavens Laboratory Animal Co., Ltd. (Animal license number: SCXKSu20160010). The mice were housed throughout the experiment in the specific pathogen-free (SPF) animal facility of Guang’anmen Hospital, maintained at a temperature of 25°C ± 1°C, a relative humidity of 55%–65%, and a 12-h light/12-h dark cycle. All mice had free access to standard rodent chow and water. Every effort was made to minimize any potential suffering of the mice. All experiments were approved by the Ethics Committee of IMPLAD (Institute of Medicinal Plant Development), CAMS & PUMC (Beijing, China, Approval NO. SLXD-20170323019), and performed under the guidance of the National Act on use of Experimental Animals (China). After a 2-week acclimation period, the *db/db* mice were randomly divided into four groups, each consisting of 12 mice. The normal control group (*db/m* mice) and the model control group mice (*db/db* + Veh) received ultrapure water (5 mL/kg). The remaining *db/db* mice were divided into two groups: one received metformin (Met) solution (0.25 g/kg, *db/db* + Met), and the other received DZF solution (0.40 g/kg, *db/db* + DZF). All drugs were administered in the form of a solution using ultrapure water as the vehicle. Mouse body weight was measured weekly, and the treatment continued for 12 weeks. At the end of the treatment period, the mice were fasted for 12 h but had access to water. Blood samples were collected under anesthesia, and liver and ileum tissues were isolated. Blood samples were left to stand at room temperature for 30 min, centrifuged at 3,000 rpm for 15 min, and the upper serum was collected and stored at −80°C. Liver and ileum tissues were either stored at −80°C or fixed in 4% paraformaldehyde.

### 2.3 Glucose oxidase method for fasting blood glucose (FBG) measurement

FBG levels were measured at weeks 0, 4, 8, and 12 after drug administration. Each measurement was initiated at the same time, with the mice subjected to a 6-h fasting period without water deprivation. The procedure involved securing the mice, disinfecting the tail tip with 75% alcohol, trimming approximately 1 mm from the tail using ophthalmic scissors, and collecting about 20 μL of blood. A cotton ball was gently applied to the tail tip to stop bleeding. After allowing the blood to stand for 30 min, it was centrifuged at 3,500 rpm for 10 min. Subsequently, 3 μL of the upper serum layer was mixed with 9 μL of ultrapure water to obtain a serum sample diluted fourfold.

A gradient dilution of a 10 mM glucose standard solution was prepared to the following concentrations: 1000, 500, 250, 125, 62.5, 31.25, and 15.625 μM. The working solution was prepared by mixing reagents R1 and R2 in a 4:1 ratio and used immediately. In a 96-well plate, 5 μL of either the serum sample or standard solution was added sequentially, and ultrapure water was used as a blank control well. Each well received 195 μL of the working solution, and the plate was shaken and incubated at room temperature for 30 min. Glucose concentrations were determined by measuring the absorbance at 550 nm using a microplate reader, and a standard curve was generated for glucose concentration calculations.

### 2.4 Insulin tolerance test (ITT)

In the 11th week of drug administration, after a 6-h fasting period, blood glucose measurements were conducted following the same method as described in Section 2.3. After the baseline blood glucose measurement at 0 min, all *db/db* mice received intraperitoneal insulin injections at a dose of 0.75 U/kg. Blood glucose levels were subsequently measured at 30-, 60-, 90-, and 120-min post-insulin injection. The area under the curve (AUC) was evaluated and calculated using GraphPad Prism Version 9.

### 2.5 Serum lipid and serum transaminase measurements

Serum samples stored at −80°C were thawed at room temperature. Subsequently, 100 μL of serum was collected and analyzed for triglyceride (TG), total cholesterol (TC), high-density lipoprotein cholesterol (HDL-C), low-density lipoprotein cholesterol (LDL-C), aspartate aminotransferase (AST), and alanine aminotransferase (ALT) levels using a fully automated clinical chemistry analyzer.

### 2.6 Measurement of liver TG and TC

Liver tissue TG were quantified using the Glycerol-3-Phosphate Oxidase-Peroxidase (GPO-PAP) enzymatic assay kit. Liver tissue samples were retrieved from −80°C storage and 100 mg of tissue was taken. These tissue samples were then added to 900 µL of pre-chilled physiological saline. A handheld electric homogenizer was used to thoroughly homogenize the tissue, followed by centrifugation at 2,500 rpm for 10 min. After centrifugation, three distinct layers were visible: cell debris at the bottom, clear liquid in the middle, and a white lipid layer on top. The clear liquid from the middle layer was collected and used for analysis. In a 96-well plate, 2.5 µL of ultrapure water (blank well), standard solution (standard well), or tissue clear liquid (sample well) were added to separate wells. Each well was then supplemented with 250 µL of working solution, and the plate was incubated at room temperature with agitation for 25 min. Absorbance was measured at a wavelength of 500 nm to determine TG concentrations. TC in the liver was determined using the Cholesterol Oxidase-Peroxidase Method (COD-PAP) reagent kit. The procedure was similar to the one described above, with absorbance measured at a wavelength of 510 nm. The protein concentration corresponding to the liver tissue clear liquid was determined using the Bicinchoninic Acid (BCA) method.

### 2.7 Histological analysis

Taking the fixed liver and ileum tissues, paraffin embedding and sectioning were performed. The sections were subjected to Hematoxylin and Eosin (H&E) staining. Optical microscopy analysis revealed cytoplasm in red and nuclei in blue. Frozen sections of liver tissue stored at −80°C were prepared and subsequently stained with Oil Red O after fixation. Optical microscopy analysis showed lipid droplets in shades of orange-red or bright red, with blue-colored nuclei.

### 2.8 16S rRNA gene sequencing

On the 12thweek, stool samples were collected and immediately stored at −80°C preparing for DNA extraction. Total microbial genomic DNA from samples of all groups was extracted with CTAB/SDS method, with the purity determined on agarose gels. The V4 region of the 16S rRNA gene was amplified with 515F-806R primers. The PCR products were purified by Agarose gel electrophoresis with GeneJET gel recovery kit (Thermo Fisher, United States). For Library preparation, Ion Plus Fragment Library Kit 48 rxns (Thermo Fisher, United States) was used. For sequencing, Life Ion S5TM or Ion S5TMXL (Thermo Fisher, United States) were used.

### 2.9 Analysis of sequencing data

The sequencing data were processed to obtain Clean Reads, and sequences with 97% identity were clustered into Operational Taxonomic Units (OTUs) by Uparse software. For species annotation analysis, Mothur method and SSUrRNA database of SILVA were used to obtain different taxonomic classifications. For alpha and beta diversity, Chao1, Shannon, UniFrac distance, UPGMA sample cluster tree was calculated or constructed using Qiime software (1.9.1). R software (2.15.3) was applied to draw the dilution, species accumulation curves, and the PCoA diagrams. The Adonis analysis was performed by R. For Linear Discriminant Analysis (LDA) Effect Size (LEfSe) analysis, LEfse software (1.0) was used with 4 as the filtering value of LDA score by default. Phylogenetic Investigation of Communities by Reconstruction of Unobserved States (PICRUSt) was adopted to predict metagenome functions of intestinal flora responded to different interventions. PICRUSt is a form of phylogenetic investigation under 16S Bioinformatics software package for metagenomic function prediction by rRNA. Detailed forecasting process to view (http://picrust.github.io/picrust/tutorials/algorithm_description.html). Comparison of PICRUST results between the two groups was performed by the *t*-test of R.

### 2.10 Measurement of serum lipopolysaccharide (LPS) levels

Frozen serum samples were thawed at room temperature. ELISA kit reagents were equilibrated to room temperature, and a gradient of standard solutions was prepared according to the manufacturer’s instructions to create the necessary working solution. Samples and standard solutions were sequentially added to a 96-well plate for the ELISA reaction. The absorbance was measured at 450 nm using a microplate reader within 5 min after adding the stop solution.

### 2.11 Western blot

Mouse liver and ileum tissues were lysed in high-efficiency RIPA lysis buffer (Beyotime, China) on ice. The tissue lysates were centrifuged at 12,000 rpm at 4°C for 15 min to obtain the protein-containing supernatant. Protein concentration was determined using the BCA protein quantification assay kit (Applygen, China), and 50 µg of protein was loaded per well for SDS-PAGE gel electrophoresis. SDS-PAGE (sodium dodecyl sulfate-polyacrylamide gel electrophoresis) gel electrophoresis was performed, and the proteins were then transferred to an activated polyvinylidene difluoride (PVDF) membrane using transfer buffer containing distilled water and methanol. The PVDF membrane was subsequently blocked with 5% skim milk and incubated with the respective primary antibodies overnight at 4°C, with β-actin serving as the internal reference protein. The membrane was then incubated with a secondary antibody conjugated with horseradish peroxidase (HRP) (Goat anti-Rabbit IgG, HRP Conjugated, Cwbio, 1:5000) at room temperature for 1 h. Enhanced chemiluminescence (ECL) plus (Beyotime, China) was applied to the membrane surface, and the membrane was placed in the Molecular Imager Gel Doc XR + System (Bio-Rad, United States) for image acquisition. Image Lab software was used to acquire images, and subsequently, ImageJ was employed for image analysis.

### 2.12 Reverse transcription-quantitative PCR (RT-qPCR)

Total RNA from mouse liver and ileum tissues was extracted using the RNAsimple Total RNA Extraction Kit (Tiangen, China). The RNA concentration was determined using a spectrophotometer (Nanodrop 2000c; Thermo Fisher, United States). cDNA synthesis was performed using the FastKing gDNA Dispelling RT SuperMix (Tiangen, China). The qPCR reaction system was prepared using SuperReal PreMix Plus (SYBR Green) (Tiangen, China), and qPCR was conducted in the qTOWER3 Real-time PCR thermal cycler (Analytik Jena, Germany). β-Actin was used as an internal control, and the relative expression of the target genes was calculated using the comparative 2^−ΔΔCT^ method. The primers corresponding to the target genes are listed in Table 1.

**TABLE 1:** Sequence of primers used for RT-qPCR

Name	Sequence(5′-3′)	
*Tjp1*	Forward	GAG​CGG​GCT​ACC​TTA​CTG​AAC
	Reverse	GTC​ATC​TCT​TTC​CGA​GGC​ATT​AG
*Ocln*	Forward	TTG​AAA​GTC​CAC​CTC​CTT​ACA​GA
	Reverse	CCG​GAT​AAA​AAG​AGT​ACG​CTG​G
*Apob*	Forward	AAG​CAC​CTC​CGA​AAG​TAC​GTG
	Reverse	CTC​CAG​CTC​TAC​CTT​ACA​GTT​GA
*Cd36*	Forward	ATG​GGC​TGT​GAT​CGG​AAC​TG
	Reverse	GTC​TTC​CCA​ATA​AGC​ATG​TCT​CC
*Fasn*	Forward	GGA​GGT​GGT​GAT​AGC​CGG​TAT
	Reverse	TGG​GTA​ATC​CAT​AGA​GCC​CAG
*Scd1*	Forward	TTC​TTG​CGA​TAC​ACT​CTG​GTG​C
	Reverse	CGG​GAT​TGA​ATG​TTC​TTG​TCG​T
*Cpt1a*	Forward	CTC​CGC​CTG​AGC​CAT​GAA​G
	Reverse	CAC​CAG​TGA​TGA​TGC​CAT​TCT
*Slc27a2*	Forward	GAT​GCC​GTG​TCC​GTC​TTT​TAC
	Reverse	GAC​TTC​AGA​CCT​CCA​CGA​CTC
*Actb*	Forward	CGT​TGA​CAT​CCG​TAA​AGA​CC
	Reverse	AAC​AGT​CCG​CCT​AGA​AGC​AC

### 2.13 Statistical analysis

Data were expressed as mean ± standard deviation (SD). Statistical analyses were performed using GraphPad Prism Version 9. Unpaired *t*-test and One-way ANOVA were used for continuous variables. Differences were considered statistically significant at *p* < 0.05.

## 3 Result

### 3.1 DZF alleviates body weight and glucose-lipid metabolic abnormalities in *db/db* mice

**FIGURE 1:**
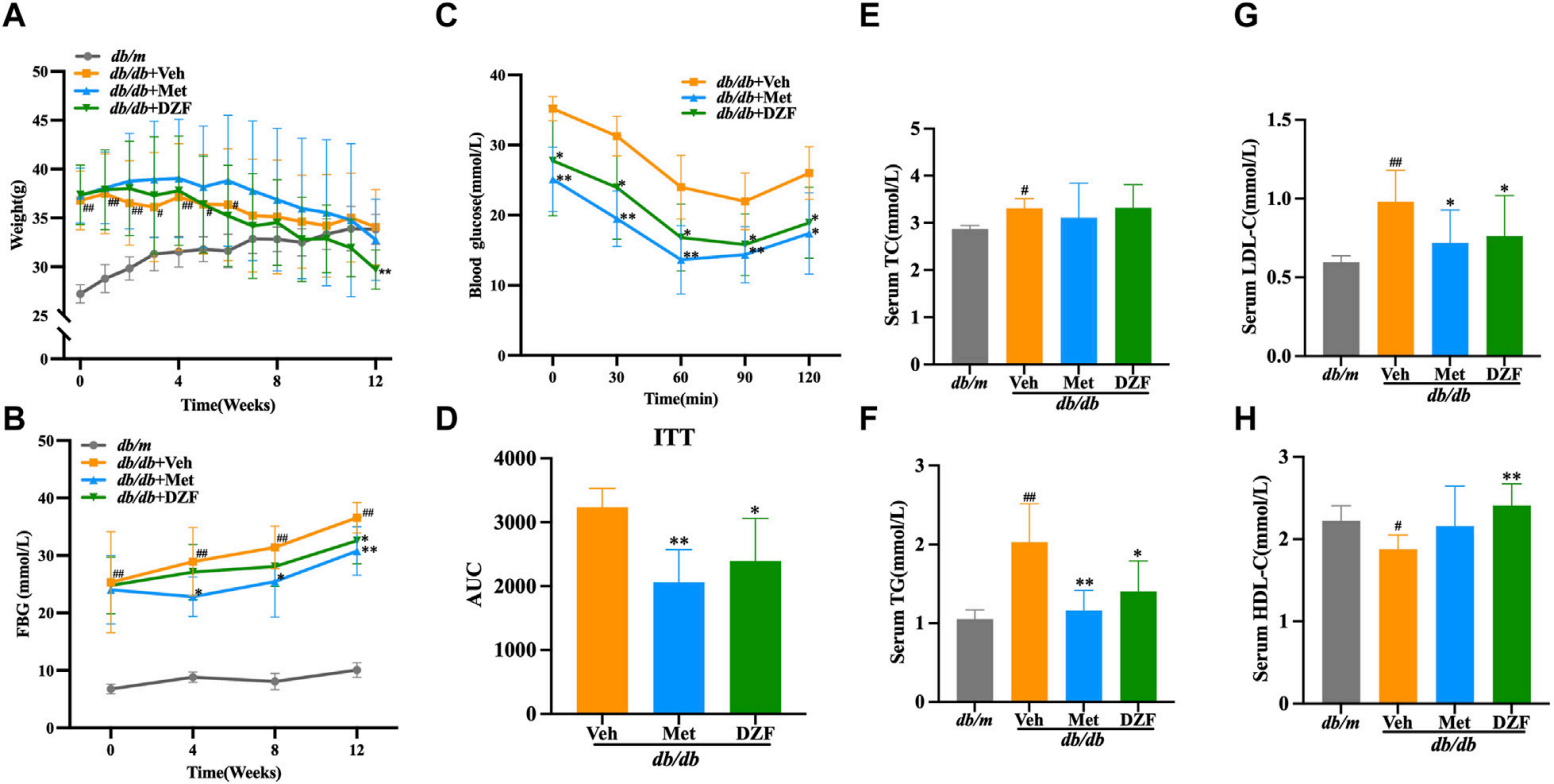
DZF alleviates body weight and glucose-lipid metabolic abnormalities in *db/db* mice. **(A)** Changes in body weight from week 0–12 of treatment. **(B)** Fasting blood glucose (FBG) levels at weeks 0, 4, 8, and 12 of treatment. **(C)** Blood glucose levels during the insulin tolerance test (ITT). **(D)** Area under the curve (AUC) during the ITT. **(E)** Serum total cholesterol (TC) levels at week 12 of treatment. **(F)** Serum triglyceride (TG) levels at week 12 of treatment. **(G)** Serum low-density lipoprotein cholesterol (LDL-C) levels at week 12 of treatment. **(H)** Serum high-density lipoprotein cholesterol (HDL-C) levels at week 12 of treatment. Data are presented as mean ± SD (A&B: n = 12, **(C)** n = 6, **(E–H)**: *n* = 10). ^#^ *p* < 0.05, ^##^ *p* < 0.01 vs. *db/m* group; ^*^ *p* < 0.05, ^**^ *p* < 0.01 vs. *db/db* + Veh group.

At the initiation of treatment, in the 8th week of age, the body weight of *db/db* mice was significantly higher than that of *db/m* group (*p* < 0.01). Although *db/db* + Veh group showed a slight decrease in body weight after the 7th week of treatment, there was no statistically significant difference in body weight at the 12th week compared to the peak body weight observed at the 6th week. Following Met intervention, a decrease in body weight in mice was observed from the 7th week onwards. While there was no statistical difference in body weight between Met and Veh groups until the end of the treatment, at the 12th week, the body weight of the Met group was significantly lower than the peak body weight observed at the 6th week (*p* < 0.05). DZF intervention led to a decrease in body weight in *db/db* mice from the 5th week of treatment. At the 12th week, the body weight in the DZF group was significantly lower than that at the peak of the 4th week (*p* < 0.01) and also significantly lower than that of the Veh group at the 12th week (*p* < 0.01) (Figure 1A).

FBG levels in *db/db* mice were significantly higher than in *db/m* mice at the start of the intervention (*p* < 0.01). Over the 12-week observation period, FBG levels in *db/m* mice remained stable, while in all three *db/db* groups, FBG levels showed a continuous upward trend. At each FBG measurement time point during the 12-week treatment, FBG in *db/db* + Veh group was significantly higher than in *db/m* group (*p* < 0.01). At weeks 4, 8, and 12, FBG in *db/db* + Met group was significantly lower than in *db/db* + Veh group, with statistical differences (*p* < 0.05 or *p* < 0.01). DZF intervention suppressed the rise in FBG levels, with a significant reduction compared to the Veh group at the 12th week (*p* < 0.05) (Figure 1B).

After 11 weeks of treatment, *db/db* mice, excluding *db/m* mice, underwent an ITT test. Met and DZF significantly reduced blood glucose levels at 30, 60, 90, and 120 min after insulin injection (*p* < 0.05 or *p* < 0.01) and significantly reduced the area under the curve (*p* < 0.05 or *p* < 0.01) (Figures 1C, D).

At 12 weeks of treatment, compared to *db/m* group, *db/db* + Veh group showed a significant increase in serum TG, TC, and LDL-C (*p* < 0.05 or *p* < 0.01), and a significant decrease in HDL-C (*p* < 0.05). Compared to *db/db* + Veh group, the Met group exhibited a significant reduction in TG and LDL-C (*p* < 0.05 or *p* < 0.01). The DZF group showed a significant reduction in TG and LDL-C (*p* < 0.05 or *p* < 0.01) and a significant increase in HDL-C (*p* < 0.01) (Figures 1E–H).

### 3.2 DZF modulates gut microbiota abundance, diversity, and metabolic function in *db/db* mice

**FIGURE 2:**
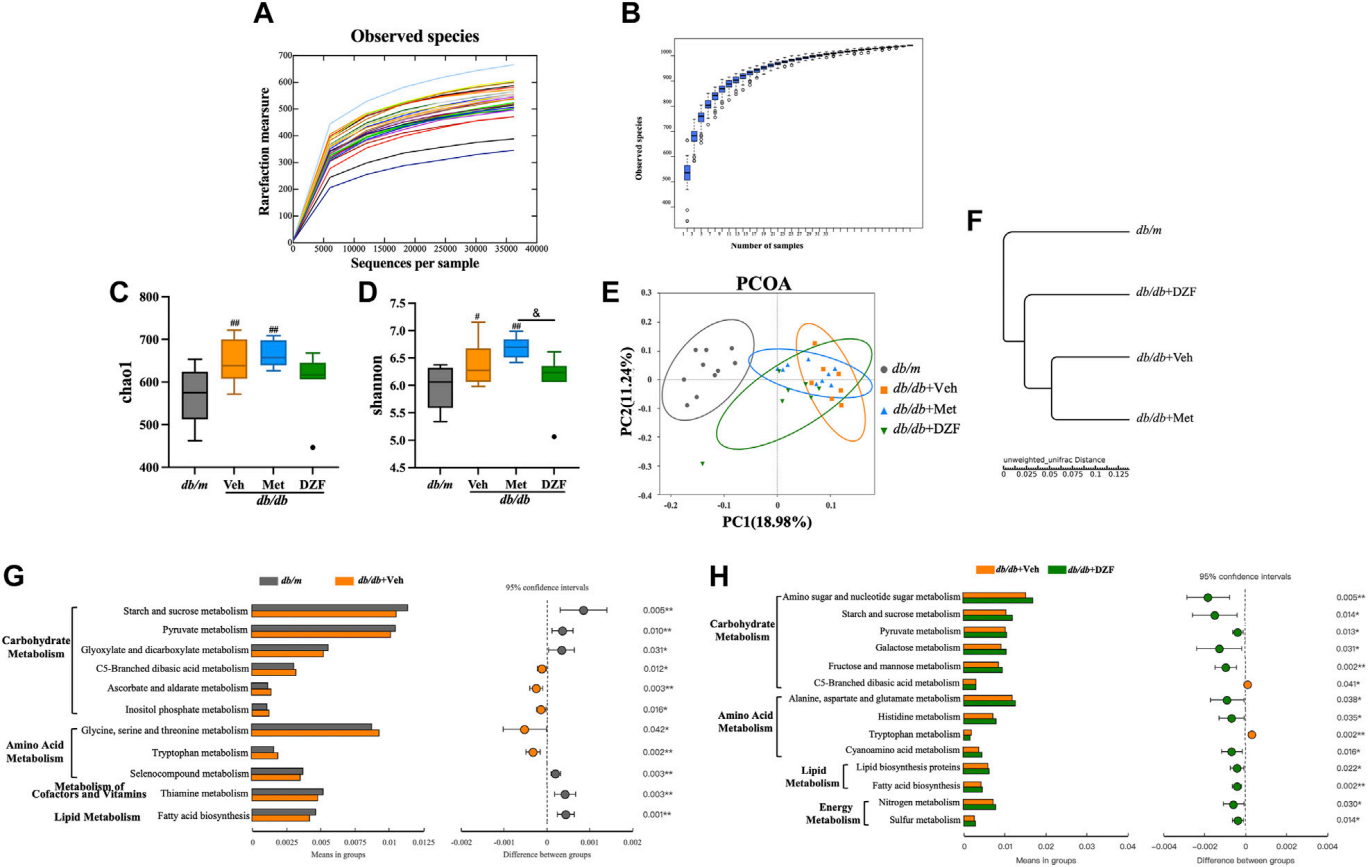
DZF modulates gut microbiota abundance, diversity, and metabolic function in *db/db* mice. **(A)** Dilution curve. **(B)** Species accumulation model. **(C)** Alpha diversity: Chao1. **(D)** Alpha diversity: Shannon. **(E)** β diversity: UniFrac distance-based unweighted principal coordinate analysis. **(F)** UPGMA clustering based on unweighted UniFrac distance. **(G)** Comparison of gut microbiota function predicted using PICRUSt between *db/m* and *db/db* + Veh groups. **(H)** Comparison of gut microbiota function predicted using PICRUSt between *db/db* + Veh and *db/db* + DZF groups. Data were presented as mean ± SD [**(A–H)**: *n* = 7]. ^#^ *p* < 0.05, ^##^ *p* < 0.01 vs. *db/m* group; ^*^ *p* < 0.05, ^**^ *p* < 0.01 vs. *db/db* + Veh group; ^&^ *p* < 0.05 vs. *db/db* + Met group.

As described in the methods, fecal samples were collected from all four groups of mice at week 12, and their microbiota composition was determined. A rarefaction curve and species accumulation model (Specaccum) analysis were employed. The observed species richness and diversity plateaued as sequencing sample size and depth increased, indicating that the current sample size and sequencing depth were sufficient to analyze community structure (Figures 2A, B). At the level of α diversity, *db/db* + Veh mice exhibited significantly increased richness and diversity compared to *db/m* mice (*p* < 0.05 or *p* < 0.01), and Met-treated *db/db* mice also displayed higher richness (*p* < 0.01) (Figures 2C, D). DZF reduced microbial diversity compared to the Met group (*p* < 0.05) (Figure 2D) but showed no significant difference compared to *db/m* mice and other *db/db* mice.

To investigate the overall differences in microbiota structure among groups, principal coordinate analysis (PCoA) based on UniFrac distances was used for β diversity analysis. The differences in PCoA were assessed using Anosim. The unweighted PCoA (Figure 2E) clearly showed a larger difference between gut microbiota of *db/m* mice and *db/db* + Veh mice (Anosim, *r* = 0.9026, *p* = 0.001). DZF intervention resulted in a significant shift compared to *db/db* + Veh mice (*r* = 0.4937, *p* = 0.001). Relative abundance at the phylum level among groups was compared using unweighted UniFrac distances (Figure 2F), suggesting that the gut microbiota of DZF-treated *db/db* mice was more similar to that of *db/m* mice. These results reflect that DZF can alter the gut microbiota composition of *db/db* mice to make it more similar to that of *db/m* mice.

Using PICRUSt based on 16S rRNA gene sequencing data and the KEGG database, the impact of DZF on gut microbiota metabolic function in *db/db* mice was predicted. Figures 2G, H mainly demonstrate differences between groups at KEGG level 2 and level 3 within the KEGG level 1 metabolism category. Differences in gut microbiota metabolic function between *db/m* and *db/db* mice primarily involved carbohydrate metabolism, amino acid metabolism, cofactors and vitamins, and lipid metabolism (*p* < 0.05 or *p* < 0.01, Figure 2G). DZF intervention could modulate carbohydrate metabolism, amino acid metabolism, lipid metabolism, and energy metabolism in *db/db* mice (*p* < 0.05 or *p* < 0.01, Figure 2H). Specifically, DZF intervention brought the predicted functional capabilities of *db/db* mouse gut microbiota in closer alignment with those of *db/m* mouse gut microbiota, including functions related to pyruvate metabolism, C5-Branched dibasic acid metabolism, tryptophan metabolism, and fatty acid biosynthesis (Figures 2G, H).

### 3.3 DZF modulates the gut microbiota composition at different taxonomic levels in *db/db* mice

**FIGURE 3:**
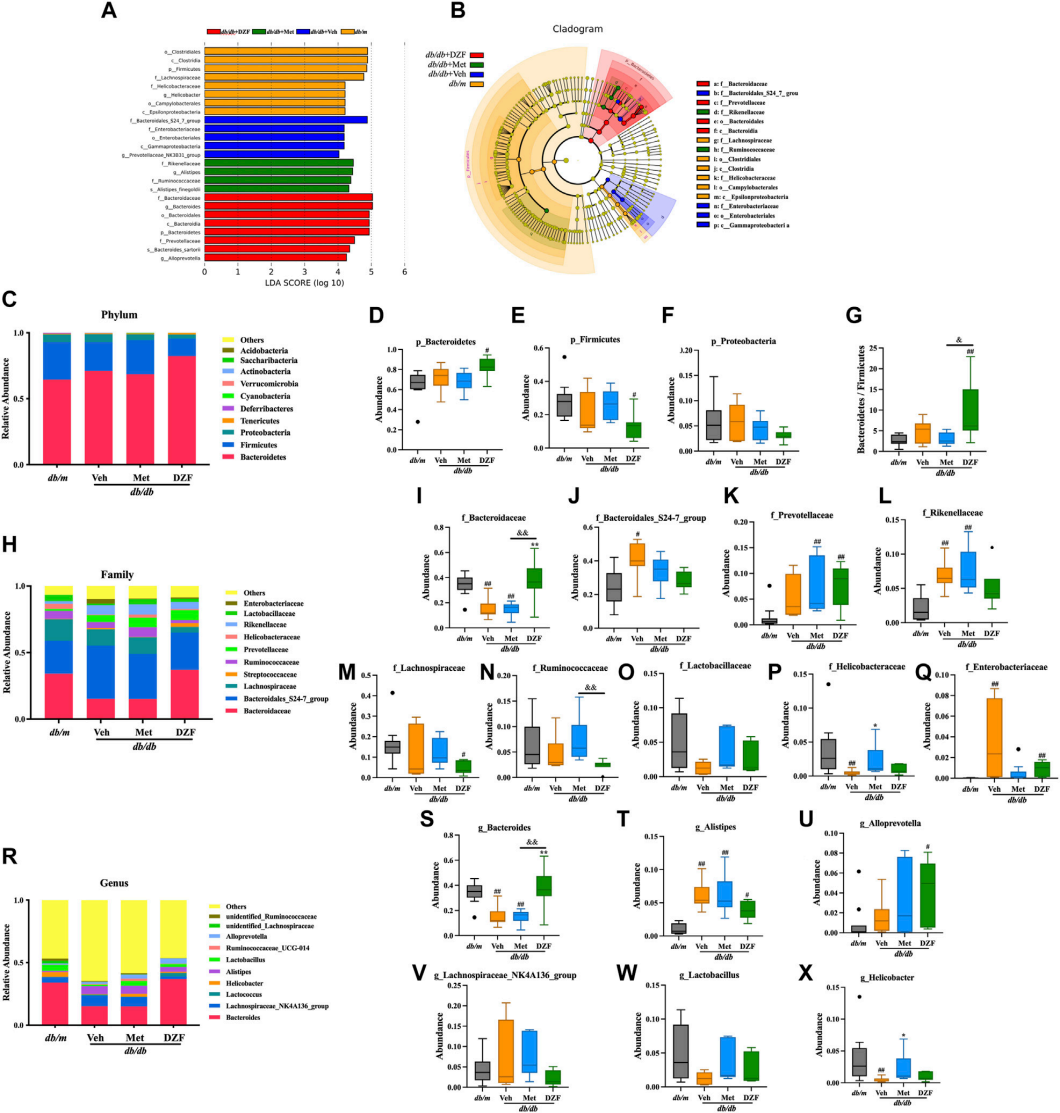
DZF modulates the gut microbiota composition at different taxonomic levels in *db/db* mice. **(A)** Distribution bar chart of LEfSe-LDA (Linear Discriminant Analysis) values: displaying species with LDA scores greater than 4, indicating significantly different biomarkers between groups. The length of the bars represents the magnitude of their influence (i.e., LDA score). **(B)**. LEfSe cladogram: a radiating circle chart representing the taxonomic hierarchy from phylum to family. Each smaller circle corresponds to a specific taxonomic level, with the size of the circles positively correlated with their relative abundance. Yellow circles represent species with no significant differences, while colored circles indicate species with differences, serving as biomarkers. **(C)** Abundance of the top 10 gut microbiota species at the phylum level. **(D–G)** Abundance of *Bacteroidetes*, *Firmicutes*, *Proteobacteria*, and the *Bacteroidetes*/*Firmicutes* ratio in *db/m* and *db/db* mice treated with vehicle, Met, and DZF. **(H)** Abundance of the top 10 gut microbiota species at the family level. **(I–Q)** Abundance of *Bacteroidaceae*, *Bacteroidales_S24-7_group*, *Prevotellaceae*, *Rikenellaceae*, *Lachnospiraceae*, *Ruminococcaceae*, *Lactobacillaceae*, *Helicobacteraceae*, and *Enterobacteriaceae* in *db/m* and *db/db* mice treated with vehicle, Met, and DZF. **(R)** Abundance of the top 10 gut microbiota species at the genus level. **(S–X**) Abundance of *Bacteroides*, *Alistipes*, *Alloprevotella*, *Lachnospiraceae_NK4A136_group*, *Lactobacillus*, and *Helicobacter* in *db/m* and *db/db* mice treated with vehicle, Met, and DZF. Data were presented as mean ± SD [**(A–X)**: *n* = 7]. ^#^ *p* < 0.05, ^##^ *p* < 0.01 vs. *db/m* group; ^*^ *p* < 0.05, ^**^ *p* < 0.01 vs. *db/db* + Veh group; ^&^ *p* < 0.05 vs. *db/db* + Met group.

To gain a deeper understanding of the characteristics of the gut microbiota, LEfSe analysis was employed to identify statistically significant biomarkers differentiating between groups. The LDA bar chart (Figure 3A) presented the key biomarkers for each group with LDA scores exceeding 4, while the accompanying cladogram (Figure 3B) illustrated the taxonomic hierarchy of these biomarkers. These biomarkers were primarily summarized from the phylum down to the family level. In the gut microbiota of *db/m* mice, biomarkers identified were *Firmicutes* phylum–*clostridia* class–*clostridiales* order–*Lachnospiraceae* family, and *Epsilonproteobacteria* class - *Campylobacterales* order–*Helicobacteraceae* family. For the gut microbiota of *db/db* + Veh group, the biomarkers identified were *Gammaproteobacteria* class–*enterobacteriales* order–*enterobacteriaceae* family, and *Bacteroidales_S24_7_group* family. In *db/db* + Met group, the identified biomarkers were *Rikenellaceae* family - *Alistipes* genus, as well as *Ruminococcaceae* family. In *db/db* + DZF group, the biomarkers included *Bacteroidetes* phylum–*Bacteroidia* class–*Bacteroidales* order-*Bacteroidaceae* family–*Bacteroides* genus, and at the same taxonomic level, *Prevotellaceae* family–*Alloprevotella* genus.

Based on the species annotation results, a bar chart was generated by selecting the top 10 species at the chosen phylum level in each group using a maximum value sorting method (Figure 3C). *Bacteroidetes* was the most abundant phylum. At the phylum level, the primary comparison focused on *Bacteroidetes*, *Firmicutes*, and *Proteobacteria*. There was no significant difference at the phylum level between *db/db* + Veh mice and *db/m* mice (Figures 3D–G). However, interestingly, DZF significantly increased the abundance of Bacteroidetes (*p* < 0.05, Figure 3D) and decreased Firmicutes (*p* < 0.05, Figure 3E) in *db/db* mice, resulting in a significant reduction in the Bacteroidetes/Firmicutes ratio (*p* < 0.01 vs. *db/m*, *p* < 0.05 vs. *db/db* + Met, Figure 3G). No significant changes were observed in the Met group compared to other groups.

At the family level, among the top 10 high-abundance taxa, *Bacteroidaceae* had the highest abundance (Figure 3H). Compared to *db/m*, both *db/db* + Veh and *db/db* + Met groups had a significant decrease in *Bacteroidaceae* (*p* < 0.01), while DZF intervention increased the abundance of *Bacteroidaceae* in *db/db* mice, significantly (*p* < 0.01) (Figure 3I). Relative to *db/m*, *db/db* + Veh group showed a significant increase in *Bacteroidales_S24-7_group* (*p* < 0.01), which slightly decreased after DZF and Met interventions but without statistical significance (Figure 3J). Interestingly, Met and DZF treatments resulted in a significant increase in *Prevotellaceae* compared to *db/m* (*p* < 0.01), while no significant difference was observed between *db/db* + Veh mice and *db/m* mice (Figure 3K). *Rikenellaceae*, along with the aforementioned three family-level groups, belongs to the *Bacteroidetes* phylum and significantly increased in *db/db* + Veh and *db/db* + Met groups (*p* < 0.01 vs. *db/m*, Figure 3L). *Lachnospiraceae*, *Lactobacillaceae*, and *Ruminococcaceae* belong to the *Firmicutes* phylum. Following DZF intervention, there was a significant reduction in the abundance of *Lachnospiraceae* compared to *db/m* + Veh group (*p* < 0.05, Figure 3M). In *db/db* + Met group, there was a tendency to increase the abundance of *Ruminococcaceae* compared to *db/db* + DZF (*p* < 0.01, Figure 3N). No significant intergroup differences were observed in the other microbial families (Figure 3O). *Proteobacteria* at the family level had *Helicobacteraceae* and *Enterobacteriaceae* as higher abundance groups. Compared to *db/m*, *Helicobacteraceae* significantly decreased in *db/db* + Veh (*p* < 0.01), while Met treatment resulted in a significant increase in *Helicobacteraceae* (*p* < 0.05, Figure 3P). Regarding *Enterobacteriaceae*, a significant increase was observed in *db/db* mice compared to *db/m* (*p* < 0.01), and DZF intervention led to a reduction in *Enterobacteriaceae* (*p* < 0.01, Figure 3Q).

At the genus level, *Bacteroides* was the most abundant genus (Figure 3R). Compared to *db/m*, both *db/db* + Veh and *db/db* + Met groups had a significant reduction in *Bacteroides* (*p* < 0.01), while DZF significantly increased its abundance relative to the Veh and Met groups (*p* < 0.01) (Figure 3S). The abundance of *Alistipes* was significantly increased in all three *db/db* mouse groups compared to *db/m* (*p* < 0.05 or *p* < 0.01, Figure 3T). Additionally, DZF treatment significantly increased the abundance of *Alloprevotella* (*p* < 0.05), while other group abundances also increased but without statistical significance (Figure 3U). *Lachnospiraceae_NK4A136_group*, *Lactobacillus*, and *Ruminococcaceae* at the genus level showed no significant intergroup differences (Figures 3V, W). The abundance of *Helicobacter* was significantly decreased in *db/db* mice compared to *db/m* (*p* < 0.01), but increased after Met treatment (*p* < 0.01) (Figure 3X).

### 3.4 DZF protects intestinal barrier and reduces intestinal lipid absorption in *db/db* mice

**FIGURE 4:**
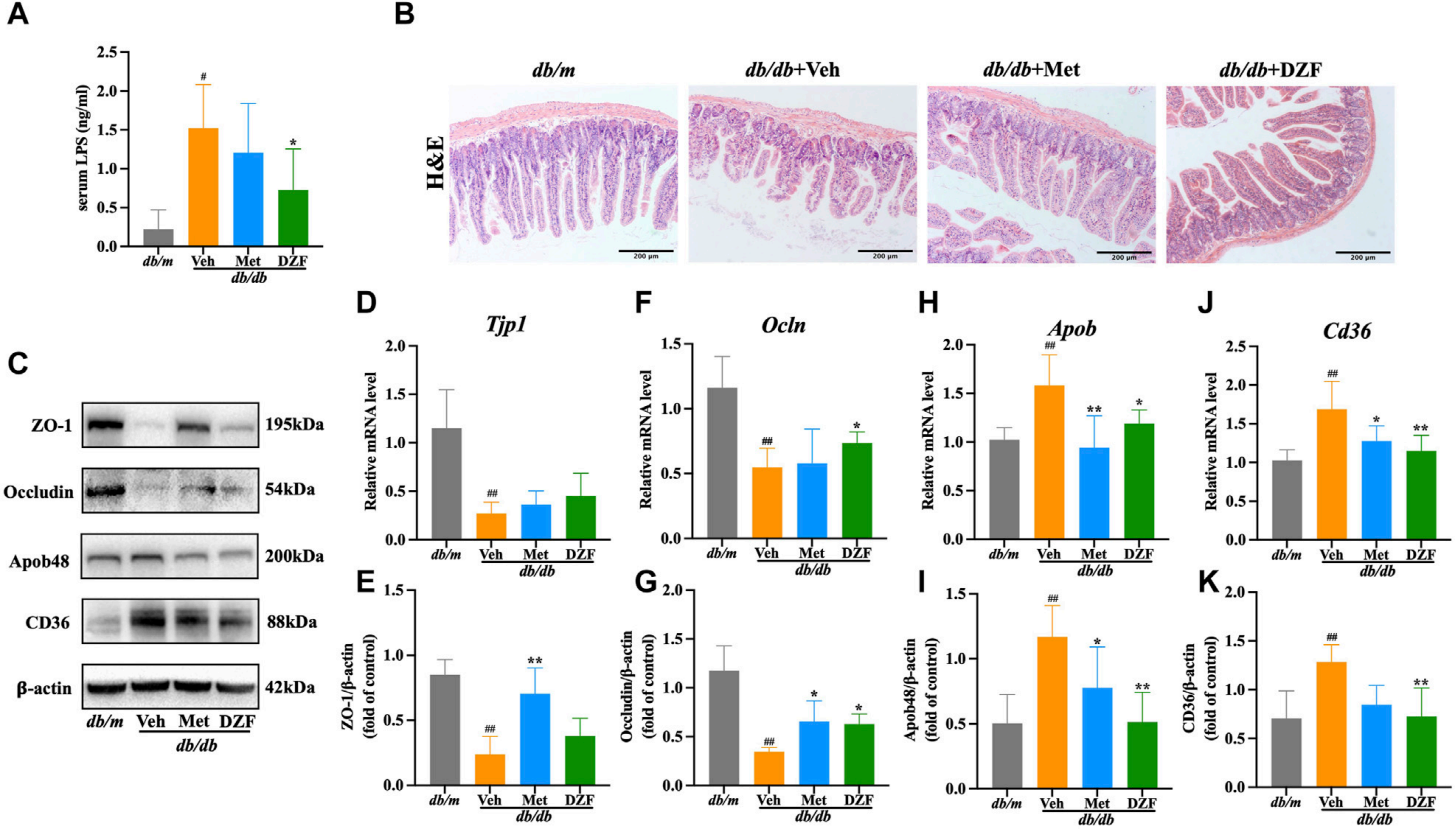
DZF protects intestinal barrier and reduces intestinal lipid absorption in *db/db* mice. **(A)** Comparison of lipopolysaccharide (LPS) concentrations in serum of *db/db* mice. **(B)** H&E staining of small intestine (×100). **(C)** Representative immunoblot images of ZO-1, Occludin, ApoB48, and CD36 in mouse small intestine. **(E,G,I,K)** Relative protein expression levels of ZO-1, Occludin, ApoB48, and CD36 in mouse small intestine. **(D,F,H,J)** Relative mRNA levels of *Tjp1*, *Ocln*, *Apob*, and *Cd36*. Data are presented as mean ± SD [**(A–K)**: *n* = 6]. ^#^ *p* < 0.05, ^##^ *p* < 0.01 vs. *db/m* group; ^*^ *p* < 0.05, ^**^ *p* < 0.01 vs. *db/db* + Veh group.

LPS, a unique component of the outer membrane of Gram-negative bacteria, also known as endotoxin, can be absorbed from the intestine into the bloodstream. In this study, it was observed that the serum LPS level in *db/db* + Veh mice was significantly higher than in *db/m* mice (*p* < 0.05). However, after DZF intervention, there was a significant reduction in LPS levels compared to the Veh group (*p* < 0.05). Met group showed a decreasing trend in LPS levels but without statistical significance (Figure 4A).

Histological examination of small intestine mucosal tissue using H&E staining revealed that in *db/m* group, the intestinal layers were well-defined, villi were neatly arranged, the intestinal wall was intact, and there was no visible damage. In contrast, in *db/db* + Veh group, the intestinal villi were shortened and deformed, with a loose and disordered arrangement. The intestinal wall showed signs of damage, and there was a reduction in the number of glands. Met and DZF interventions both led to a partial restoration of the structural integrity of the small intestine. Villi length increased, gaps narrowed, intestinal wall damage reduced, and the number of glands increased (Figure 4B).

Zonula Occludens-1 (ZO-1) and Occludin, known as tight junction proteins, are critical components of the intestinal mechanical barrier. In *db/db* + Veh group, the expression of ZO-1 and Occludin protein was significantly lower than in *db/m* group (*p* < 0.01). The expression of *Tjp1* and *Ocln* mRNA of *db/db* + Veh group was also significantly lower than *db/m* group (*p* < 0.01). Compared to *db/db* + Veh group, Met significantly upregulated the expression of ZO-1 and Occludin proteins (*p* < 0.05 or *p* < 0.01), with no significant difference in *Tjp1* and *Ocln* mRNA levels. Compared to *db/db* + Veh group, DZF intervention significantly upregulated Occludin protein and *Ocln* mRNA expression (*p* < 0.05), while it had no significant impact on ZO-1 expression (Figures 4C–G).

Cluster of differentiation 36 (CD36) is a key protein in intestinal epithelial cells responsible for fatty acid absorption, while apolipoprotein B48 (ApoB48) is a crucial protein for assembling chylomicrons to transport lipids in intestinal cells. In *db/db* + Veh group, the expression of CD36 and ApoB48 protein was significantly higer than in *db/m* group (*p* < 0.01). The expression of *Cd36* and *Apob* mRNA of *db/db* + Veh group was also significantly higer than *db/m* group (*p* < 0.01). This result indicates abnormal elevation in intestinal lipid absorption and transport. Compared to *db/db* + Veh, DZF intervention significantly downregulated the expression of CD36 protein, ApoB48 protein, *Cd36* mRNA and *Apob* mRNA (*p* < 0.05 or *p* < 0.01). Compared to *db/db* + Veh, Met intervention significantly downregulated ApoB48 protein and *Apob* mRNA expression (*p* < 0.05 or *p* < 0.01) and reduced *Cd36* mRNA expression (*p* < 0.05), with no statistically significant difference in CD36 protein expression (Figures 4C, H–K).

### 3.5 DZF reduces hepatic steatosis in *db/db* mice

**FIGURE 5:**
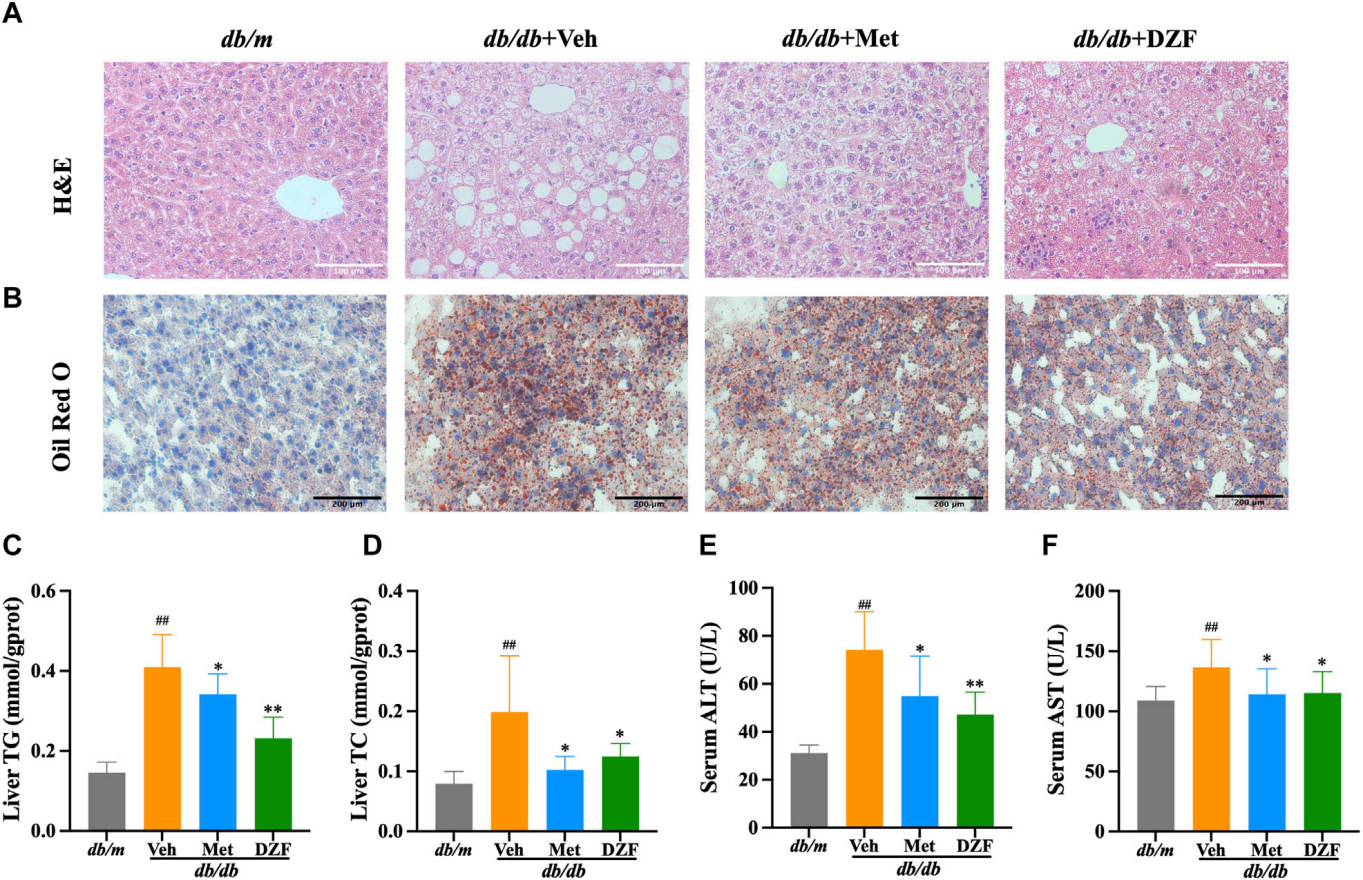
DZF reduces hepatic steatosis *db/db* mice. **(A)** Hepatic H&E staining (×200). **(B)** Hepatic Oil Red O staining (×100). **(C)** Hepatic triglyceride (TG) content in mice. **(D)** Hepatic total cholesterol (TC) content in mice. **(E)** Serum alanine aminotransferase (ALT). **(F)** Serum aspartate aminotransferase (AST). Data were presented as mean ± SD **(A,B)**: *n* = 6; **(C–F)**: *n* = 10). ^#^ *p* < 0.05, ^##^ *p* < 0.01 vs. *db/m* group; ^*^ *p* < 0.05, ^**^ *p* < 0.01 vs. *db/db* + Veh group.

Hepatic tissue morphology was observed through H&E staining. In *db/m* group, liver tissue displayed intact structure, orderly cell arrangement, well-defined hepatic cords, centrally located cell nuclei, and pink cytoplasm without signs of lipid vacuole degeneration or significant inflammatory cell infiltration. In *db/db* + Veh group, liver cells were swollen, arranged irregularly, and showed signs of lipid vacuole degeneration, with increased accumulation of lipid droplets, varying in size. Some nuclei were shifted towards the periphery of the cell, and the cytoplasm appeared pale or transparent. In *db/db* + Met group, there was a moderate reduction in lipid vacuole degeneration, with smaller vacuoles being predominant. The liver cells displayed less swelling and more orderly arrangement, with cytoplasm appearing light pink. In *db/db* + DZF group, the improvement in lipid deposition was more pronounced, with fewer liver cells exhibiting lipid vacuole degeneration. Some liver cells were arranged in a more regular pattern, with nuclei centrally located (Figure 5A).

Further examination of lipid droplet distribution within liver cells was performed using Oil Red O staining. In *db/m* group, liver cells were neatly arranged, without noticeable orange-red lipid droplets inside the cells. The nuclei were blue and uniformly sized. In *db/db* + Veh group, a significant number of irregularly sized orange-red lipid droplets were observed, with liver cells arranged more chaotically. In *db/db* + Met and *db/db* + DZF groups, the volume and quantity of orange-red lipid droplets within liver cells were reduced, indicating a decrease in the degree of lipid deposition (Figure 5B).

Hepatic TG and TC levels in *db/db* + Veh group were significantly higher than those in *db/m* group (*p* < 0.01), indicating severe hepatic steatosis in *db/db* mice. Compared to *db/db* + Veh, both DZF and Met interventions led to a reduction in hepatic TC and TG to varying degrees (*p* < 0.05 or *p* < 0.01), with DZF showing a more significant decrease in TG (Figures 5C, D). Compared to *db/m*, serum ALT and AST levels were significantly elevated in *db/db* mice, indicating liver cell damage due to hepatic steatosis. However, after DZF and Met interventions, ALT and AST levels significantly decreased (*p* < 0.05 or *p* < 0.01), indicating that DZF and Met could protect liver function (Figures 5E, F).

### 3.6 DZF regulates hepatic lipid metabolism pathways in *db/db* mice

**FIGURE 6:**
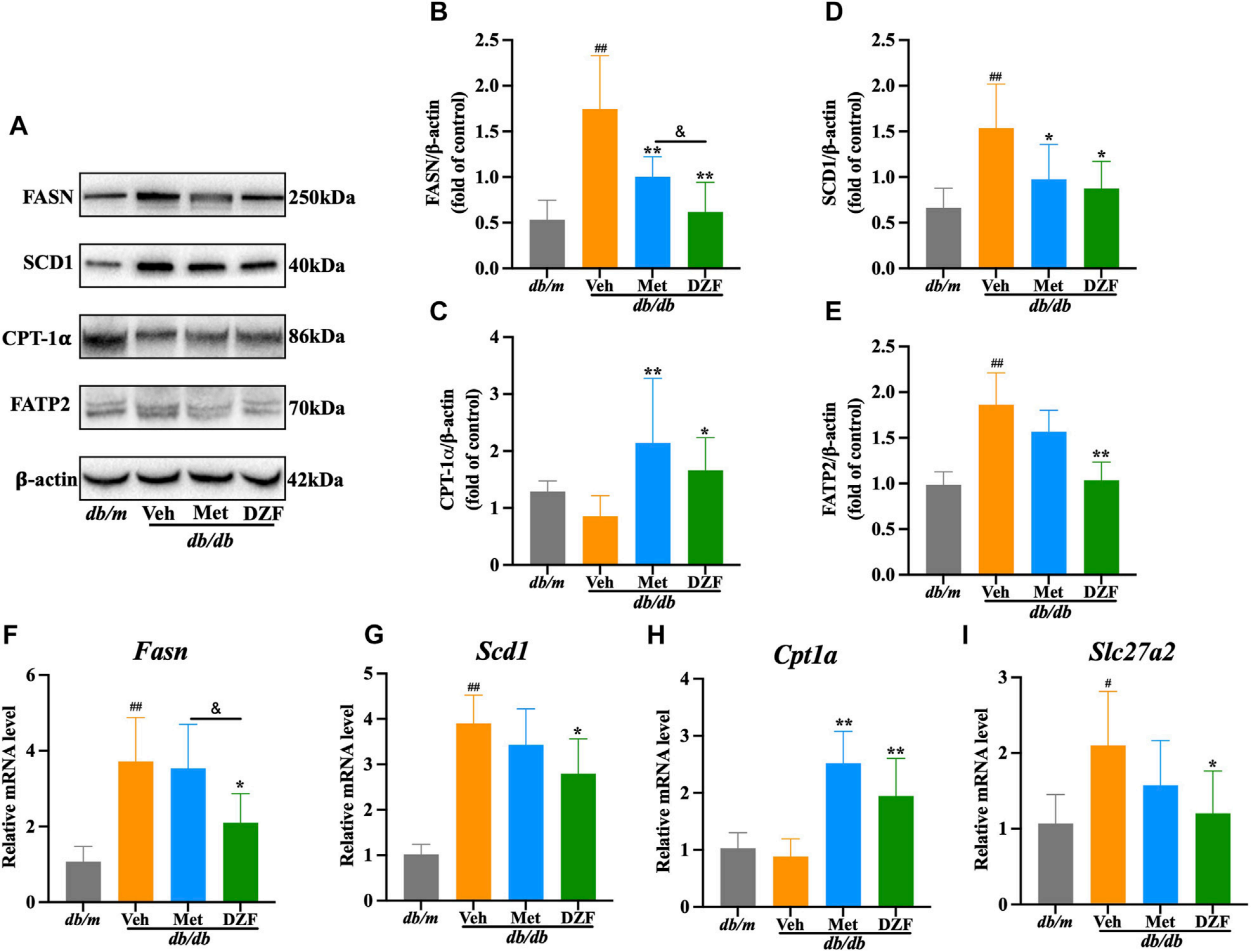
DZF regulates hepatic lipid metabolism pathways in *db/db* mice. **(A)** Representative immunoblot images of FASN, SCD1, CPT-1𝛂, and FATP2 in mouse liver. **(B–E)** Relative protein expression levels of FASN, SCD1, CPT-1𝛂, and FATP2 in mouse liver. **(F–I)** Relative mRNA levels of *Fasn*, *Scd1*, *Cpt1a*, and *Slc27a2*. Data are presented as mean ± SD [**(A–K)**: *n* = 6]. ^#^ *p* < 0.05, ^##^ *p* < 0.01 vs. *db/m* group; ^*^ *p* < 0.05, ^**^ *p* < 0.01 vs. *db/db* + Veh group.

Fatty acid synthase (FASN) and stearoyl-CoA desaturase 1 (SCD1) are key enzymes in hepatic fatty acid synthesis. In comparison to *db/m* mice, the expression of FASN protein, SCD1 protein, *Fasn* mRNA and *Scd1* mRNA in the liver of *db/db* mice were significantly elevated (*p* < 0.01). Met intervention led to a decrease in protein expression of FASN and SCD1 (*p* < 0.05 or *p* < 0.01), with a corresponding decrease in *Fasn* and *Scd1* mRNA expression, although without statistical significance. In contrast, DZF exhibited a more pronounced inhibitory effect on fatty acid synthesis. Compared to *db/db* + Veh group, *db/db* + DZF group showed a significant reduction in the expression of FASN protein, SCD1 protein, *Fasn* mRNA and *Scd1* mRNA (*p* < 0.05 or *p* < 0.01). Furthermore, the FASN protein and *Fasn* mRNA expression in *db/db* + DZF group were significantly lower than that in *db/db* + Met group (*p* < 0.05) (Figures 6A, B, D, F, G).

Carnitine palmitoyltransferase-1𝛂 (CPT-1𝛂) is a major enzyme responsible for fatty acid oxidation in the liver. Both *db/db* and *db/m* mice showed similar and not significantly different CPT-1𝛂 protein and *Cpt1a* mRNA expression levels. Following DZF and Met interventions, CPT-1𝛂 protein and *Cpt1a* mRNA expression was significantly increased (*p* < 0.05 or *p* < 0.01), indicating enhanced fatty acid oxidation in liver cells (Figures 6A, C, H).

Fatty acid transport protein 2 (FATP2) mediates the uptake of fatty acids by liver cells. In *db/db* + Veh group, the expression of FATP2 protein and *Slc27a2* mRNA was significantly increased (*p* < 0.05 or *p* < 0.01). After DZF intervention, a decrease in the expression of FATP2 protein and *Slc27a2* mRNA was observed (*p* < 0.05 or *p* < 0.01), indicating that DZF can inhibit the excessive uptake of fatty acids by liver cells. Although there was a decreasing trend with Met intervention, no statistically significant differences were observed (Figures 6A, E, I).

## 4 Discussion

Hepatic lipid metabolism is a crucial component of the complex metabolic network in the body. Hepatic steatosis is not only a result of overall metabolic abnormalities but also an exacerbating factor for metabolic dysfunction. Numerous studies have demonstrated the substantial potential and advantages of TCM in improving metabolism. Derived from classical Chinese medical formulas, DZF has significantly ameliorated weight, blood sugar, and lipid abnormalities in MetS mice in multiple studies. Moreover, it has alleviated hepatic fat deposition morphologically (Zhu et al., 2018; Huang et al., 2022a). These effects were reaffirmed in this study. Due to the close anatomical location and functional relationship, hepatic metabolism is intricately linked to the influence of the gastrointestinal tract. This study, therefore, examines the potential mechanisms by which DZF affects hepatic lipid metabolism from this perspective.

The intestine is a critical organ influencing liver metabolism, and the complex interactions between them are collectively referred to as the gut-liver axis (Hsu and Schnabl, 2023). Within the intestine, a vast community of microorganisms profoundly and extensively impacts metabolic diseases such as obesity, type 2 diabetes, and hepatic steatosis (de Vos et al., 2022). In this study, the gut microbiota of *db/db* mice exhibited higher α-diversity compared to *db/m* mice. Although it is generally accepted that α-diversity in gut microbiota is significantly reduced in human with obesity (Wilmanski et al., 2019), there are still research findings and reviews that have drawn opposite conclusions, or some studies that have not observed significant differences (Lippert et al., 2017; Chávez-Carbajal et al., 2019; Gong et al., 2022). Multiple studies utilizing *db/db* mice as an animal model have reported findings consistent with the observations in this study (Liu et al., 2019; Wang et al., 2020; Tu et al., 2022), suggesting that the results of this study may reflect the genuine characteristics of *db/db* mice. However, this phenomenon is not yet fully understood and may require consideration of differences at specific taxonomic levels. Following DZF intervention, α diversity displayed a declining trend, becoming more similar to that of normal mice. From a β diversity perspective, there were significant differences in the gut microbiota community structure between *db/db* and *db/m* mice, while DZF-treated *db/db* mice exhibited a gut microbial community composition more similar to that of *db/m* mice.

Upon further taxonomic examination, representative microbial groups in *db/db* mice belong to *Enterobacteriaceae*. *Enterobacteriaceae* are facultative anaerobes with a wide pathogenic potential (Shelton and Byndloss, 2020). Increased *Enterobacteriaceae* levels can lead to an inflammatory state, resulting in impaired intestinal epithelial function, increased intestinal permeability, and LPS biosynthesis, which are observed in conditions like inflammatory bowel disease and diabetes (Pedersen et al., 2018; Andoh and Nishida, 2023). DZF intervention shifted the gut microbiota to make *Bacteroidetes* the dominant microbiota and elevated the *Bacteroidetes*/*Firmicutes* (B/F) ratio. *Firmicutes* and *Bacteroidetes* are the two most abundant phyla at the mammalian gut microbiota level, with the B/F ratio varying in different diseases. Several studies on obesity have observed a reduction in *Bacteroidetes* and an increase in *Firmicutes* (Lee and Lee, 2020). DZF displayed a more significant upregulation of *Bacteroidetes*, consistent with the trends in the effects of berberine, the primary extract from Huanglian, on the gut microbiota of obese mice (Habtemariam, 2020). *Bacteroidetes* have various beneficial effects and contribute to improving host metabolism and immune homeostasis. Studies have reported that centenarians have *Bacteroidetes*-dominant gut microbiota (Pang et al., 2023). *Bacteroidetes* can produce less toxic LPS, which help reduce LPS-induced inflammatory responses (Magne et al., 2020; Zafar and Saier, 2021). Following DZF intervention, there is a decrease in serum LPS levels, and an increase in the expression of intestinal epithelial cell tight junction proteins, indicating that DZF can alleviate the intestinal barrier damage caused by gut microbiota imbalance.

Furthermore, a decrease in the expression of CD36 and ApoB48 was observed following DZF intervention. CD36 is a crucial membrane protein responsible for identifying and transporting fatty acids (Cifarelli and Abumrad, 2018). ApoB48 is responsible for intracellular lipid assembly into chylomicrons (Ko et al., 2020). DZF increased the abundance of the *Bacteroidetes*, which is known to produce inositol and sphingolipids extensively, thereby regulating intestinal lipid metabolism (Johnson et al., 2020; Heaver et al., 2022). This suggests that DZF can alleviate the excessive activation of lipid absorption and transport functions in the intestine. The intestinal mucosal barrier and nutrient absorption are critical functions of the intestinal mucosa. Lipid absorption predominantly occurs in the small intestine and is closely associated with the intestinal barrier. Lipids can permeate through the brush border’s permeable barrier or be taken up by intestinal cells through proteins like CD36 and fatty acid-binding proteins (FABP) (Sallee and Dietschy, 1973; Xu et al., 2021). Furthermore, intestinal lipid absorption and intestinal barrier function are jointly regulated by multiple pathways (Wu et al., 2022). For instance, the activation of intestinal group 3 innate lymphoid cells (ILC3) can efficiently optimize and control the efficiency of lipid absorption in the intestine while safeguarding the intestinal barrier (Talbot et al., 2020). Studies have shown that berberine, a major active component of DZF, significantly increases intestinal ILC3 and regulates intestinal immune homeostasis (Dong et al., 2022), suggesting a potential mechanism by which DZF regulates intestinal absorption and barrier functions.

Intestinal barrier dysfunction and abnormalities in lipid absorption are well-known risk factors for metabolic disorders such as hepatic steatosis and insulin resistance. Dietary habits play a significant role in shaping the gut microbiota, which, in turn, influences intestinal mucosal function. When the barrier is compromised, it can lead to increased lipid absorption, resulting in elevated levels of circulating lipids and endotoxins. On the one hand, excessive lipid deposition causes a decrease in insulin sensitivity of adipose tissue and an increase in lipolysis, a phenomenon known as “lipotoxicity” (Guilherme et al., 2008). On the other hand, pathogen-associated molecular patterns (PAMPs), including LPS, can activate pattern recognition receptors (PRRs) in the liver via the portal vein, such as Toll-like receptors (TLRs), leading to the secretion of proinflammatory cytokines. This initiates chronic low-grade inflammation, exacerbating insulin resistance and lipid metabolism abnormalities and potentially causing hepatocyte damage (Louis-Jean and Martirosyan, 2019; Vallianou et al., 2021). These pathological changes affect various aspects of hepatic lipid metabolism, resulting in an imbalance between the influx and efflux of lipids. Hepatic steatosis fundamentally stems from an increase in both *de novo* lipogenesis and the uptake of circulating free fatty acids, as well as a reduction in fatty acid oxidation and lipid output (Lee et al., 2023). In our study, DZF intervention in *db/db* mice led to a significant reduction in key enzymes related to *de novo* hepatic lipid synthesis, namely FASN and SCD1, as well as in the protein FATP2 responsible for fatty acid transport. Additionally, the enzyme CPT-1𝛂, associated with fatty acid oxidation, showed a significant increase. DZF demonstrated its regulatory effect on hepatic lipid generation, uptake, and consumption, thereby ameliorating systemic glucose and lipid metabolism abnormalities and preserving hepatic function. These findings suggest that DZF may serve as a potential therapeutic approach for hepatic steatosis caused by metabolic disorders. Our previous research has shown that DZF can improve insulin resistance in the liver and skeletal muscles of *db/db* mice, reduce serum non-esterified fatty acids, and exhibit anti-metabolic disorder effects (Zhu et al., 2018). At the same time, it has been observed that DZF regulates the gut microbiota, improves intestinal barrier function, and enhances lipid absorption. This is based on the profound mutual influence within the gut-liver axis, suggesting that the gut microbiota and mucosal function are potential targets for DZF in ameliorating hepatic lipid metabolism abnormalities.

Non-alcoholic fatty liver disease (NAFLD) is the most prevalent chronic liver disease at present. Its pathological manifestation involves hepatic steatosis affecting more than 5% of liver cells, accompanied by or without lobular inflammation and hepatocyte ballooning. NAFLD is closely associated with MetS (Pafili and Roden, 2021). The latest international consensus suggests renaming NAFLD as metabolic dysfunction-associated fatty liver disease (MAFLD), emphasizing the significant impact of metabolic disorders on hepatic steatosis (Eslam et al., 2020). There are currently no approved treatments for NAFLD (Powell et al., 2021). Our research results indicate that DZF effectively alleviates MetS-associated hepatic steatosis. This suggests that DZF has the potential to be a therapeutic agent for NAFLD, although further research is needed for confirmation.

Metabolites closely related to the gut microbiota also play a crucial role in the gut-liver axis, such as short-chain fatty acids (SCFA) and bile acids (BA) (Ringseis et al., 2020; Collins et al., 2023). SCFAs originate from food and are metabolically produced by the gut microbiota, playing a role in regulating overall energy homeostasis and glucose-lipid metabolism. Following DZF intervention, there is a significant increase in the abundance of beneficial bacterial genera, such as *Bacteroides*, *Alistipes*, and *Alloprevotella*, which are known to promote SCFA production (Li et al., 2021a; Zou et al., 2023). Predictive results from PICRUSt suggest that DZF can upregulate the lowered carbohydrate metabolism levels of *db/db* mice. Microbial carbohydrate metabolism is involved in the pathogenesis of host insulin resistance (Takeuchi et al., 2023), with carbohydrates, especially pyruvate, playing a crucial role in the generation of SCFAs (Vervier et al., 2022). This suggests that SCFAs are a potential target for the action of DZF. However, further research is required to understand how DZF utilizes SCFAs to improve hepatic lipid metabolism. Bile acids (BAs) are produced during cholesterol metabolism in the liver and promote the absorption of dietary lipids in the intestines. After metabolic modification by the gut microbiota, the majority of BAs are reabsorbed back into the liver through the enterohepatic circulation (Fuchs and Trauner, 2022). BAs also serve as important signaling molecules, with the farnesoid X receptor (FXR) being one of their ligands. FXR is highly expressed in both the intestines and the liver and is beneficial for intestinal barrier function and hepatic lipid metabolism. Currently, FXR agonists such as obeticholic acid are potential drugs for treating NAFLD and have entered the clinical trial phase (Simbrunner et al., 2021). Research has shown that berberine can alter the BA profile in NAFLD mice and increase FXR expression (Wang et al., 2021), suggesting that BAs could be one of the potential targets for DZF in regulating the gut-liver axis. Our research team is actively investigating the mechanisms by which DZF affects BA metabolism and the FXR-related pathways.

## 5 Conclusion

**FIGURE 7:**
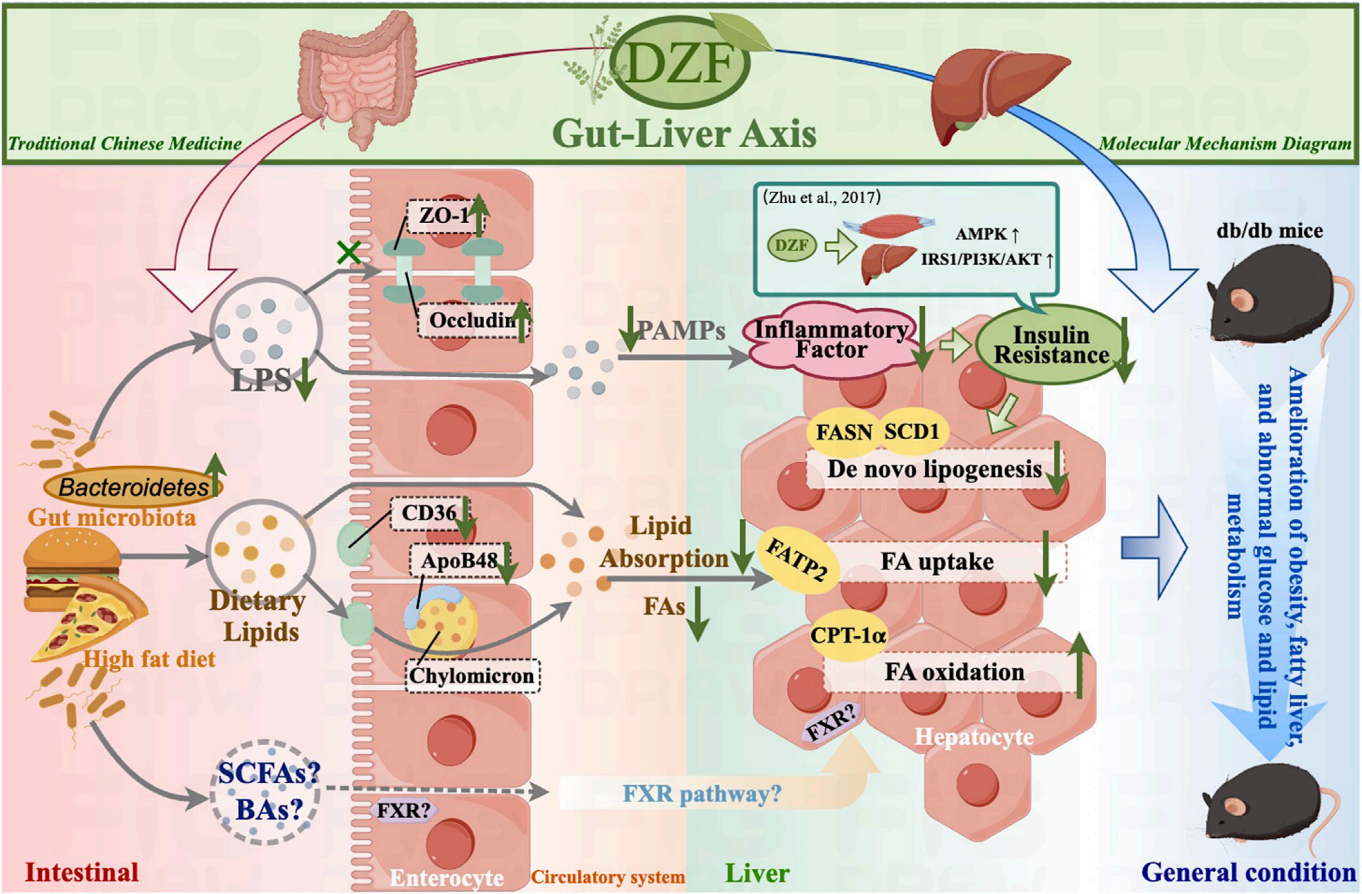
Summary diagram of DZF molecular mechanisms in this study. DZF: Dai-Zong-Fang, LPS, lipopolysaccharide; SCFA, short-chain fatty acid; BA, bile acid; FA, fatty acid; PAMP, pathogen-associated molecular pattern. (Pictrue by Figdraw).

In conclusion, DZF may improve intestinal barrier and lipid absorption function by modulating the gut microbiota, thus alleviating hepatic steatosis and systemic metabolic abnormalities (Figure 7). The molecular mechanisms through which DZF improves metabolism via the gut-liver axis are worthy of further in-depth investigation, thus providing pharmacological evidence for the use of DZF in the treatment of metabolic conditions such as MetS and fatty liver.

## Figures and Tables

**Table:** 

**ALT**	alanine aminotransferase
**ApoB48**	apolipoprotein B48
**AST**	aspartate aminotransferase
**BA**	bile acid
**BCA**	bicinchoninic acid
**CD36**	cluster of differentiation 36
**CPT-1𝛂**	carnitine palmitoyltransferase-1𝛂
**DZF**	Dai-Zong-Fang
**FASN**	fatty acid synthase
**FATP2**	fatty acid transport protein 2
**FBG**	fasting blood glucose
**FXR**	farnesoid X receptor
**HDL-C**	high-density lipoprotein cholesterol
**ILC3**	intestinal group 3 innate lymphoid cells
**ITT**	insulin tolerance test
**LDA**	linear discriminant analysis
**LDL-C**	low-density lipoprotein cholesterol
**LEfSe**	LDA Effect Size
**LPS**	lipopolysaccharide
**MetS**	metabolic syndrome
**NAFLD**	non-alcoholic fatty liver disease
**OTUs**	Operational Taxonomic Units
**PICRUSt**	Phylogenetic Investigation of Communities by Reconstruction of Unobserved States
**SCD1**	stearoyl-CoA desaturase 1
**SCFA**	short-chain fatty acid
**TC**	total cholesterol
**TCM**	Traditional Chinese Medicine
**TG**	Triglyceride
**ZO-1**	Zonula Occludens-1

## Data Availability

The data presented in the study are deposited in the NCBI Sequence Read Archive, BioProject accession number PRJNA1064673.
